# The Black Yeast *Exophiala dermatitidis* and Other Selected Opportunistic Human Fungal Pathogens Spread from Dishwashers to Kitchens

**DOI:** 10.1371/journal.pone.0148166

**Published:** 2016-02-11

**Authors:** Jerneja Zupančič, Monika Novak Babič, Polona Zalar, Nina Gunde-Cimerman

**Affiliations:** 1 Department of Biology, Biotechnical Faculty, University of Ljubljana, Ljubljana, Slovenia; 2 Centre of Excellence for Integrated Approaches in Chemistry and Biology of Proteins (CIPKeBiP), Ljubljana, Slovenia; Louisiana State University, UNITED STATES

## Abstract

We investigated the diversity and distribution of fungi in nine different sites inside 30 residential dishwashers. In total, 503 fungal strains were isolated, which belong to 10 genera and 84 species. Irrespective of the sampled site, 83% of the dishwashers were positive for fungi. The most frequent opportunistic pathogenic species were *Exophiala dermatitidis*, *Candida parapsilosis sensu stricto*, *Exophiala phaeomuriformis*, *Fusarium dimerum*, and the *Saprochaete/Magnusiomyces* clade. The black yeast *E*. *dermatitidis* was detected in 47% of the dishwashers, primarily at the dishwasher rubber seals, at up to 10^6^ CFU/cm^2^; the other fungi detected were in the range of 10^2^ to 10^5^ CFU/cm^2^. The other most heavily contaminated dishwasher sites were side nozzles, doors and drains. Only *F*. *dimerum* was isolated from washed dishes, while dishwasher waste water contained *E*. *dermatitidis*, *Exophiala oligosperma* and *Sarocladium killiense*. Plumbing systems supplying water to household appliances represent the most probable route for contamination of dishwashers, as the fungi that represented the core dishwasher mycobiota were also detected in the tap water. Hot aerosols from dishwashers contained the human opportunistic yeast *C*. *parapsilosis*, *Rhodotorula mucilaginosa* and *E*. *dermatitidis* (as well as common air-borne genera such as *Aspergillus*, *Penicillium*, *Trichoderma* and *Cladosporium*). Comparison of fungal contamination of kitchens without and with dishwashers revealed that virtually all were contaminated with fungi. In both cases, the most contaminated sites were the kitchen drain and the dish drying rack. The most important difference was higher prevalence of black yeasts (*E*. *dermatitidis* in particular) in kitchens with dishwashers. In kitchens without dishwashers, *C*. *parapsilosis* strongly prevailed with negligible occurrence of *E*. *dermatitidis*. *F*. *dimerum* was isolated only from kitchens with dishwashers, while *Saprochaete/Magnusiomyces* isolates were only found within dishwashers. We conclude that dishwashers represent a reservoir of enriched opportunistic pathogenic species that can spread from the dishwasher into the indoor biome.

## Introduction

Over the last two decades, the incidence of fungal infections of humans has increased at an alarming rate, which already today presents a major challenge to health care systems. This correlates with the increased average age, the large number of people with treatable chronic diseases, the growing population of immunocompromised individuals, and the practices of intensive chemotherapy and use of immunosuppressive drugs [1,2,3]. Although *Candida albicans* and *Aspergillus* spp. are the most frequently encountered human opportunistic fungal pathogens, non-albicans *Candida* spp. (e.g., *Candida glabrata*, *Candida krusei*, *Candida parapsilosis*, *Candida dubliniensis*) and different *Fusarium* spp. are also increasing in importance as emerging fungal pathogens. Indeed, 80% of all human fungal infections are caused by members of the *Fusarium solani* species complex (FSSC) and the *Fusarium oxysporum* species complex (FOSC) [1–4].

Humans are exposed to fungi outdoors and indoors. As people spend more than 80% of their time indoors on average [5], many studies have examined the relationships between housing conditions and air-borne fungi, and how these relate to respiratory symptoms, such as asthma and fungal-related allergies [6–9]. Much less is known about potential risks associated with the presence of fungi in water-related parts of households such as bathrooms [10–12] and kitchens [13–15]. The few studies on the distribution of fungi in residential kitchens have focussed on contamination with outdoor air-borne fungi [16–18], and on contamination of bathrooms and kitchen sinks by fungal biofilms in plumbing systems [13–15,19].

There have been reports of increased frequency and relative abundance of *Fusarium* spp. and *Exophiala* spp. taxa, which are known for their thermo tolerance, acid tolerance, oligotrophicity, and ability to metabolize surfactants [10–12]. Recent studies have showed that fungi also invade extreme kitchen environments, such as dishwashers [20,21]. Conditions inside dishwashers not only enable selected fungal species to survive, but also promote the selective enrichment of particular polyextremotolerant fungi that can tolerate low and high pH, temperatures up to 60°C or 80°C, occasional dehydration and high organic loads, high concentrations of NaCl, and mechanical stress due to water ejectors. The human opportunistic pathogenic black yeast *Exophiala dermatitidis* and *Exophiala phaeomuriformis* have been detected in 35% of dishwashers examined around the world [20]. Other opportunistic pathogenic species that are consistently present are *C*. *parapsilosis sensu stricto*, *Aureobasidium melanogenoum*, *Fusarium dimerum* and *Magnusiomyces capitatus* [20,21].

Fungi that inhabit dishwashers may be released into the kitchen via aerosols and waste water, and by direct contact between contaminated surfaces and hands. Thus, dishwashers are possible sources of fungal contamination and subsequently infections. An especially endangered group are immunocompromised patients with cystic fibrosis, who are often infected with *Aspergillus fumigatus*, *C*. *albicans*, *C*. *glabrata*, *C*. *parapsilosis*, *E*. *dermatitidis* and *Fusarium* spp. [22–26].

Dishwashers have been investigated to date only for the presence of fungi on rubber seals [20, 21], while their distribution inside the dishwashers and their transfer into the kitchen environment has not been investigated. In the present study, we focussed on the presence of human opportunistic fungi in 9 locations within 30 randomly chosen household dishwashers and in the tap-water system connected to these dishwashers. Additionally, we examined the spread of these fungi into the kitchen via waste water, hot dishwasher aerosols, and contact with different surfaces and dishwasher-washed dishes.

## Materials and Methods

### Sampling

**Fig 1:**
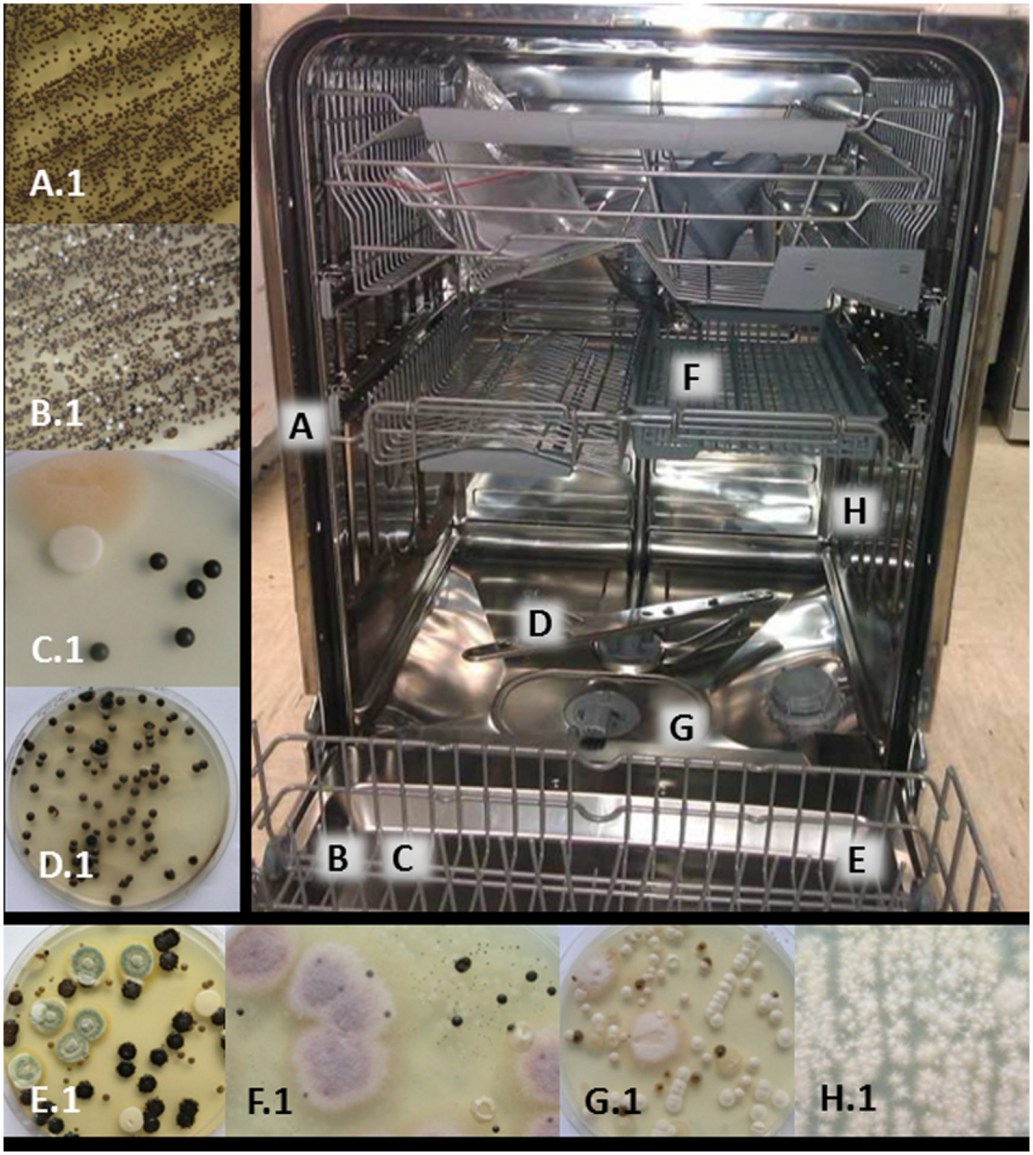
Sampling sites in the dishwashers (A-H) and the prevailing taxa on the primary isolation plates (A.1-H.1) cultured from the particular dishwashers sampling sites swab samples. On rubber seal **(A)** and detergent dispenser **(B)** *E*. *dermatitidis* genotype A **(A.1, B.1,** respectively**)**; on rinse-aid dispenser **(C)** *E*. *dermatitidis* genotype A, FDSC, *Saprochaete/Magnusiomyces* clade **(C.1)**; on sprinkler **(D)** *E*. *dermatitidis* genotype A, *E*. *phaeomuriformis* genotype 1 **(D.1)**; on door **(E)** *A*. *melanogenum*, *P*. *crustosum*, *E*. *dermatitidis* genotype A, *C*. *parapsilosis* **(E.1)**; on cutlery rack **(F)** FOSC, *E*. *phaeomuriformis* genotype 1, *C*. *parapsilosis* **(F.1)**; in drain **(G)** *E*. *dermatitidis* genotype A, *Saprochaete/ Magnusiomyces* clade, *Meyerozyma guilliermondii*, FDSC **(G.1)**; on side nozzles **(H)** FDSC **(H.1)**. Note: not the actual state of one dishwasher, but a combination of several sampled dishwashers.

In the autumn of 2013, 30 randomly selected dishwashers used in residential kitchens in seven Slovenian cities were sampled (Ljubljana, Velenje, Žalec, Celje, Mislinja, Sežana, and Portorož). The dishwasher age (length of use) was from 1 year to 8 years, they were produced by four different manufacturers, and they had different frequencies of use (i.e., from once a week, to twice per day) and cleaning processes (e.g., chemical, mechanical). Nine user-accessible sites inside each dishwasher were sampled: rubber seal, door, interior side wall, cutlery rack, detergent dispenser, rinse-aid dispenser, dishwasher drain, side nozzle, and upper and lower sprinklers (Fig 1). The internal parts of a single dishwasher that are difficult to access by the user but are in contact with water were also sampled: the water inlet tube, temperature sensor, water purity sensor, circulation pump, waste water pump, ion exchanger, hydraulic tank, and waste-water pipe. Sampling for fungi was performed immediately after a washing cycle had finished, using sterile cotton swabs moistened with physiological saline. The swabs were stored in sterile tubes at 4°C, and processed within 3 days.

For metagenomic analyses, biofilms from the rubber seals of the same 30 dishwashers were sampled by scraping the surface of the rubber seals using a sterile scalpel. The collected biomass was placed into a sterile tube and stored at -20°C. Additionally, the biofilm was also sampled from a single dishwasher that had not been used for a year and had been stored in a cool, dry place and not connected to the water supply system, using the same technique.

**Fig 2:**
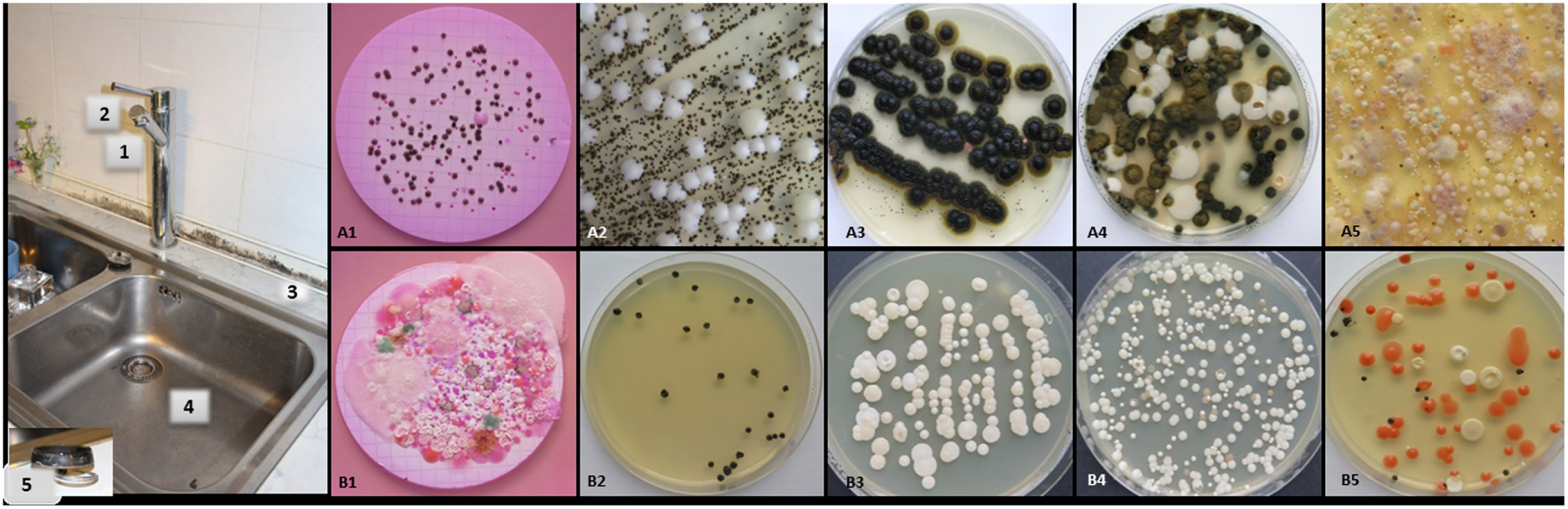
Sampling sites in the kitchens with a dishwasher (A) and the kitchens without a dishwasher (B). **(S)** Representative kitchen sink indicating sampling sites of 1 L tap water (filtered through 0.45-μm cellulose membrane filter and incubated on DRBC agar) (1), pipe grid on tap (2), kitchen counter above dishwasher/ near sink (3), and inside kitchen sink (4). Inset (5): Rubber seal from under kitchen sink. **(A, B)** Representative primary isolation plates cultured from kitchen samples, numbered according to sample site. **(A1)** *A*. *melanogenum*, *E*. *alcalophila*, *R*. *similis* and *C*. *parapsilosis*. **(B1)** *Trichosporon montevideense*, *R*. *mucilaginosa*, *C*. *parapsilosis* and *E*. *phaeomuriformis* genotype 1. **(A2)** *E*. *phaeomuriformis* genotype 1 and *C*. *parapsilosis*. **(B2)** *E*. *phaeomuriformis* genotype 1. **(A3)** *A*. *melanogenum*, *E*. *phaeomurifomis* genotype 2 and *R*. *mucilaginosa*. **(B3)** *C*. *parapsilosis*, *M*. *guilliermondii*, *C*. *pararugosa* and *P*. *kluyveri*. **(A4)** *O*. *constricta*, *Cysosporidium slooffiae* and *Metschnikowia pulcherrima*. **(B4)** *M*. *guilliermondii*, *C*. *pararugosa* and *S*. *cerevisiae*. **(A5)** *C*. *pararugosa*, *C*. *parapsilosis*, *E*. *dermatitidis* genotype A and FOSC. **(B5)** *M*. *guilliermondii*, *C*. *parapsilosis*, *C*. *orthopsilosis K*. *europaea*, *R*. *mucilaginosa*, *E*. *phaeomuriformis* genotype 1 and *A*. *melanogenum*.

Sampling for fungi was performed in the same 30 kitchens that contained the sampled dishwashers, and additionally in 14 kitchens without dishwashers. Samples were taken using sterile cotton swabs, as described above, from the following places: counter above the dishwasher (kitchens with dishwasher), or counter in the vicinity of the sink (kitchens without dishwashers), kitchen sink, drain, rubber seal on drain, grid on tap, and dish-drying rack (Fig 2).

Dishwasher-cleaned items of different materials (e.g., ceramic, porcelain, glass, stainless steel, Teflon) and forms (e.g., plates, cups, glasses, cutlery, pans) were sampled immediately after the end of dishwashing cycles at 40°C and 60°C, using sterile cotton swabs. The dishes were still warm and wet. The same types of dishes were sampled in kitchens without dishwashers, directly after hand washing.

Five litres of tap water were taken from each of the 30 sampled kitchens, and 100 mL of waste water was collected after dishwashing in seven selected dishwashers. For all of the sampled dishwashers, 100 L of released hot aerosols were sampled immediately after the termination of a washing cycle, directly onto dichloran rose bengal chloramphenicol (DRBC) agar (Oxoide, UK), as five replicates using an air sampler (SAS Super 180; Cherwell, UK). In one case, the airborne microorganisms from 1 m^3^ of hot steam at a flow rate of 100 L/min were collected (Coriolis cyclone collector; Bertin Technologies, SaintQuentin-en-Yvelines, France) in collection liquid (AES Chemunex, Bruz, France) with an initial volume of 15 mL.

### Isolation of fungi

For the samples taken from surfaces, the cotton swabs were rubbed over the surface of malt extract agar (Oxoid, Hampshire, UK) supplemented with 0.05 g/L chloramphenicol, which were then incubated at 30°C and 37°C, for up to 7 days.

For the air samples, the DRBC culture media subjected to air sampling were incubated at 25°C and 37°C, and monitored daily for fungal growth, for up to 14 days.

For the water samples, 1-L tap water samples were filtered through 0.45-μm membrane filters (Merck, Millipore). The filters were placed on DRBC agar, and were incubated at 30°C and 37°C, for up to 7 days.

Pure cultures were isolated and have been deposited at the Ex Culture Collection of Extremophilic Fungi, a part of the Infrastructural Centre Mycosmo (MRICUL) at the Department of Biology, Biotechnical Faculty, University of Ljubljana, Slovenia.

### DNA extraction

Pure fungal cultures were transferred to fresh malt extract agar medium, and after 3 days to 7 days incubation, the DNA was extracted. For yeast-like strains, PrepMan Ultra reagent (Applied Biosystems) was used, according to the manufacturer instructions. DNA extraction of filamentous fungi and *Exophiala* strains was done according to Gerrits van den Ende and de Hoog (1999) [27], after mechanical lysis of 1 cm^2^ of mycelium. Total genomic DNA extraction of all of the biofilm samples (hydrated and dehydrated) was carried out using MoBio PowerBiofilm kits (Carlsbad, CA, USA), according to the manufacturer instructions. Genomic DNA of an air sample from the collection liquid was extracted using MoBio PowerWater^®^ DNA isolation kits (Carlsbad, CA, USA). The extraction followed the manufacturer instructions, with slight modifications: 15 mL water samples were filtered through 0.45-μm membrane filters (Merck, Millipore) and placed directly into the beading tube provided in the kits. No additional modifications were introduced.

### Molecular characterisation of DNA

Identification of the yeast was based on amplification and sequencing of the large subunit ribosomal DNA sequences (LSU; partial 28S rDNA, D1/D2 domains), using the NL1 and NL4 primer set [28]. A fragment of the rDNA that included internal transcribed spacer (ITS) region 1, 5.8S rDNA, and ITS2 was amplified and sequenced for identification of *Exophiala* spp. and other filamentous fungi, using the ITS5 and ITS4 primer set [29]. For identification of *Aspergillus* and *Penicillium* strains, the partial β-tubulin gene (*benA*) was amplified and sequenced with the T1 and T22 primers [30]. *Fusarium* strains were identified using nuclear translation elongation factor 1-alpha (*tef*) sequences, amplified with the EF1 and EF2 primers [31].

The ITS, LSU, *benA* and *tef* nucleotide sequences were determined by direct PCR sequencing, performed by Microsynth AG, Switzerland. BigDye terminator cycle sequencing kits were used in the sequence reactions (Applied Biosystems, Foster City, CA, USA). The sequences were obtained using an ABI Prism 3700 Big Dye Sequencer (Applied Biosystems). The sequences were assembled using FinchTV 1.4 (Geospiza, PerkinElmer, Inc.), and automatically and manually aligned using the Molecular Evolutionary Genetics Analysis (MEGA) software, version 6.06 [32]. The assembled DNA sequences were examined using the BLAST software of the National Centre for Biotechnology Information (NCBI) database, and were compared to the appropriate sequences of the reference and type strains.

The genotypes of *E*. *dermatitidis* were defined from sequences obtained according to Matos et al. (2003) [33]. Determination of *E*. *phaeomuriformis* genotypes was made based on Zalar et al. (2011) [20].

Validation of the presence of *E*. *dermatitidis* from genomic DNA in an air sample was conducted by PCR amplification with the specific primers Eder1F (5´-TCT GGT CGA GCG TTT CCG-3´) and Eder1R (5´-AAA CCG ATA CGT GCT CAG TGA-3´), using an established protocol designed by Novak Babič et al. (2013) [34].

### Total DNA processing and bioinformatic analysis

An artificial community DNA template was obtained by combining aliquots of the 30 individual genomic DNA extracts from fresh biofilms. The amount of DNA of this representative biofilm DNA sample that was used for downstream sequencing was 50 ng. A single sample of total DNA was extracted from a dried biofilm, with an amount of 3.6 ng. Equal amount of template DNA was used for PCR reaction in both samples. Both samples were sequenced separately, where ITS2 of the nuclear ribosomal coding cistron was targeted with the ITS3 and ITS4 primers [29]. The amplicon was sequenced by Microsynth AG (Balgach, Switzerland) using the Roche 454 pyrosequencing platform. The sequences were processed bioinformatically using QIIME [35]. Reads with less than 60% similarity to any sequence in the reference dataset were discarded. Sequences with quality below 25 or shorter than 250 bp were removed. Chimeric sequences were identified using the UCHIME algorithm [36], and were discarded. The linker and reverse primer sequences were trimmed. The maximum allowed number of homopolymers in a single fungal sequence was set to eight. All of the sequences were clustered into Operational Taxonomic Units (OTUs) based on their sequence similarities. This was carried out by subsampled open reference clustering against the dynamic reference set of the UNITE database (version 6). When the sequences were clustered at 97% sequence similarity, those belonging to the same cluster were interpreted as the same species. The OTUs were identified by similarity to reference sequences (minimum similarity, 97%), and *de-novo* by clustering the remaining sequences with each other (minimum similarity, 97%). The set of representative sequences was assigned to taxonomic identities using the UNITE/QIIME ITS reference OTUs. The reference sequence set represented a clustered version (sequence similarity, 97%) of all of the fungal rDNA ITS sequences in the current UNITE+INSD databases (International Nucleotide Sequence Databases: NCBI, EMBL, DDBJ).

### Quantification of *Exophiala* spp.

The *Exophiala* spp. cell numbers per 1 cm^2^ of rubber seal surface were determined on a sample from an 8-year-old dishwasher in daily use. The samples were taken by rubbing 1 cm^2^ of rubber seal using sterile cotton swabs moistened with sterile physiological saline. The samples were processed immediately, as follows: 3 mL sterile physiological saline solution was added into the sterile tubes containing the cotton swab, and the tubes were vortexed for 1 min at maximum speed. The procedure was carried out as three technical replications. The samples were diluted in 10-fold steps to 10^−8^. A 100-μl aliquot of the undiluted samples and of each dilution was inoculated onto malt extract agar supplemented with 0.05 g/L chloramphenicol. The total CFU (Colony-forming units) were determined by colony counting after 7 days of incubation at 37°C.

### Quantification of *Exophiala* spp. during the washing cycle

To determine the number of *Exophiala* spp. cells released into the water during the washing cycle, we collected samples from the water that flowed over the dishes and from the bottom of the dishwasher drain, using collection tubes placed inside the dishwashers. These samples were removed 30, 60 and 90 min from the beginning of the washing cycle. For quantification of *Exophiala* spp., 50 mL of each water sample was filtered through 0.45-μm membrane filters (Merck, Millipore), which were then placed on DRBC medium. Black colonies were counted after 7 days of incubation at 37°C. The identity of *Exophiala* was determined after isolation and ITS sequencing, as described above.

## Results

Across all of the sampling carried out in the present study, a total 503 strains of fungi were obtained, which belong to 10 genera and 84 species, with the full data summary given in Table 1 [37]. Additionally, 3 strains of green algae lacking chlorophyll were obtained, belonging to the species *Prototheca wickerhamii*. Two algal strains were isolated from dishwasher’s detergent dispenser and one from dish drying rack inside kitchen with dishwasher.

**Table 1:** Diversity and frequency of the fungi isolated in the present study from tap water, waste water, aerosols, dishwashers and kitchen surfaces.

Compartment	Tap water		Dishwasher waste water		Dishwasher		Kitchens with dishwasher		Kitchens without dishwasher		Cleaned items		Air		Assumed entry route*
	(n)	(%)	(n)	(%)	(n)	(%)	(n)	(%)	(n)	(%)	(n)	(%)	(n)	(%)	
Total samples	44	100	7	100	30	100	30	100	14	100	44	100	29	100	
Samples with fungi	37	84	5	71	25	83	29	96	14	100	8	18	29	100	
Samples with black yeasts	16	36	5	71	19	63	22	73	9	64					
Negative samples	7	16	2	29	5	17	1	4			36	82			
**Species**															
*Acremonium* sp.							3	10							a
*Aspergillus* sp. ^1^													24	83	a
*Aspergillus* section *Fumigati*					1	3			4	29	1	2			a
*Aspergillus* section *Nigri*									4	29	1	2			a
*Aspergillus* section *Versicolores*									2	14	1	2			a
*Aureobasidium melanogenum*	7	16			2	7	7	23	2	14					w
*Aureobasidium pullulans*									2	14					a
*Candida albicans*							3	10							h
*Candida californica*							1	3							f
*Candida inconspicua*					1	3									u
*Candida glaebosa*	1	2													w
*Candida intermedia*	1	2			2	7	4	13							f, h
*Candida orthopsilosis*									3	21					h
*Candida parapsilosis*	5	11			10	33	23	77	9	64	1	2	4	14	h, w
*Candida pararugosa*	1	2					6	20	10	71					u
*Candida tropicalis*							1	3							h
*Candida zeylanoides*					1	3	1	3							u
*Capronia* sp.	1	2													w
*Cladophialophora boppii*							1	3							u
*Cladosporium* sp.					2	7	1	3			1	2	6	21	a
*Clavispora lusitaniae*					2	7	4	13							u
*Cryptococcus albidus*					1	3	2	7							h, f
*Cryptococcus diffluens*							2	7							u
*Cryptococcus liquefaciens*					3	10	1	3							h
*Cryptococcus saitoi*									1	7					h, f
*Cystobasidium slooffiae*	3	7			1	3	2	7	2	14					w
*Debaryomyces carsonii*							1	3							u
*Debaryomyces hansenii*					1	3	2	7							f
*Exophiala alcalophila*	1	2													w
*Exophiala dermatitidis*	1	2	3	43	14	47	7	23	1	7			1	3	w
*Exophiala lecanii-corni*	3	7					1	3	2	14					w
*Exophiala mesophila*							1	3							w
*Exophiala oligosperma*			2	29	1	2									w
*Exophiala phaeomuriformis*	4	9			9	30	11	37	7	50					w
*Exophiala spinifera*							1	3							u
*Exophiala xenobiotica*									1	7					u
*Fonsecaea* sp.							1	3							u
FDSC	3	7			7	23	5	17			1	2			w
FOSC					4	13	10	33	4	29	1	2			a
FSSC					2	7	3	10							u
*Geotrichum candidum*	1	2					2	7							f
*Hanseniaspora uvarum*							2	7							f, w
*Kluyveromyces marxianus*							1	3							f
*Knufia epidermidis*									1	7					u
*Kwoniella europaea*									7	50					u
*Lecythophora* sp.									2	14					u
*Lichtheimia* sp.							1	3							u
*Lodderomyces elongisporus*							1	3							f
*Metschnikowia fructicola*					1	3	1	3							u
*Metschnikowia pulcherrima*							1	3							f
*Meyerozyma caribbica*	1	2													f, h, w
*Meyerozyma guilliermondii*					7	23	18	60	10	71					f, h
*Mucor circinelloides*							1	3							f
*Myrothecium* sp.							1	3							f
*Myrothecium verrucaria*							1	3							f
*Ochroconis constricta*							4	13	3	21					w
*Penicillium crustosum*					1	3									a
*Penicillium* sp.					2	7	2	7			1	2	16	55	a
*Phoma* sp.									1	7	1	2			f, a, w
*Pichia burtonii*							1	3							f
*Pichia cactophila*							1	3							u
*Pichia kluyveri*							1	3	3	21					f
*Pichia kudriavtevii*					2	7									f
*Pichia membranifaciens*									2	14					f
*Pichia onychis*							1	3							u
*Pseudozyma* sp.	2	5					1	3							w
*Rhodosporidium diobovatum*					1	3									w
*Rhodosporidium kratochvilovae*					1	3									w
*Rhinocladiella similis*	3	7													w
*Rhodotorula minuta*									3	21					w
*Rhodotorula mucilaginosa*	1	2			7	23	21	70	11	79	1	2	2	7	w, f, h, a
*Saccharomyces cerevisiae*					2	7	7	23	8	57					f
*Saprochaete/Magnusiomyces* clade					8	27									u
*Sarocladium killiense*			1	14											w
*Schizophyllum commune*	1	2													w
*Sporobolomyces* sp.									1	7					
*Talaromyces verruculosus*	1	2					1	3							w
*Trametes versicolor*					1	3									a
*Trichoderma* sp.											1	2	6	21	a
*Trichosporon montevideense*	1	2													w
*Trichosporon ovoides*							2	7							h
*Trichosporonoides* sp.							1	3							u
*Wickerhamomyces anomalus*							2	7							h
*Yarrowia lipolytica*							2	7							u

^1^ Not identified to species level.

FDSC *Fusarium dimerum* species complex; FOSC *Fusarium oxysporum* species complex; FSSC *Fusarium solani* species complex.

* Assumed route of entry (summarised from The yeasts, a taxonomic study [37]; different ecology chapters): a, air; w, water; f, food remains; h, human inhabitants; p, pets; u, unknown.

### Water as the vector for fungal contamination of household appliances

Plumbing systems that supply the water to household dishwashers represent the most probable route for contamination of appliances with fungi. Therefore, 30 tap-water samples from kitchens with dishwashers and 14 from kitchens without dishwashers were tested for fungi (Table 1). Out of 44 samples, 84% (37/44) were positive for fungi, with levels from 1 CFU/L to >300 CFU/L. In total, 41 fungal strains were isolated that belong to 8 genera and 19 species (Table 1). For these water samples, according to the fungal content of the relevant dishwasher, there were no differences in the fungal compositions. The predominant species was the black yeast *A*. *melanogenum* (16%; 7/44), with a range from 1 CFU/L to 35 CFU/L, followed by the black yeast *Rhinocladiella similis* (7%; 3/44) and *E*. *phaeomuriformis* (9%; 4/44). *E*. *dermatitidis* was isolated in only 2% of samples (1/44), while 10% were positive for other *Exophiala* species, including *Exophiala alcalophila* (1/44) and *Exophiala lecanii-corni* (3/44). *C*. *parapsilosis* was detected in 11% (5/44) of water samples, while all of the other representatives of non-parapsilosis species were present in 6% of samples (3/44). *C*. *albicans* was not detected in these tap-water samples. *R*. *mucilaginosa* and *Cystobasidium slooffiae* together were isolated from 2% (1/44) and 6% (3/44) of the water samples, *Pseudozyma crassa* and the FDSC were both found in 5% (2/44) and 7% (3/44), respectively, of the samples, whereas other fungal species were isolated only sporadically.

### Fungi colonise the interior of dishwashers

The sampling of nine sites in 30 randomly selected dishwashers showed that irrespective of the sampling site, 83% (25/30) of the tested dishwashers were positive for fungi (Fig 1). In total, 97 fungal strains were isolated that belong to 10 genera and 29 species (Table 1). Furthermore, many of the individual sampling sites showed multiple contaminations, with the isolation of up to 5 different fungi from a single sampling site per dishwasher (Table 2). The most frequently detected species were *E*. *dermatitidis*, *C*. *parapsilosis sensu stricto*, *E*. *phaeomuriformis*, *Saprochaete/Magnusiomyces* clade and the *F*. *dimerum* species complex (FDSC) (Tables 2 and 3).

**Table 2:** Frequency of occurrence of strains and their sampling sites and GenBank accession numbers from the 30 residential dishwashers sampled in the present study.

Location	Positive dishwasher	Identification mode	Representative strain—EXF No.	GenBank Accession No.
Identification				
**Rubber seal**				
*Candida intermedia*	1	LSU	EXF-9640	KP761089
*Candida parapsilosis*	6	LSU	EXF-9283	KP761091
*Exophiala dermatitidis* genotype A	8	ITS	EXF-9512	KP761128
*Exophiala dermatitidis* genotype A2	1	ITS	EXF-9510	KP761135
*Exophiala dermatitidis* genotype B	2	ITS	EXF-9286	KP761136
*Exophiala dermatitidis* genotype C	1	ITS	EXF-9227	KP761141
*Exophiala oligosperma*	1	ITS	EXF-9647	KP761144
*Exophiala phaeomuriformis* genotype 1	4	ITS	EXF-8884	KP761145
FDSC	1	ITS	EXF-9155	KP761153
FOSC	4	*tef*	EXF-9270	KP761169
FSSC	1	*tef*	EXF-9331	KP761172
*Meyerozyma guilliermondii*	3	LSU	EXF-9689	KP761104
*Rhodosporidim diobovatum*	1	LSU	EXF-9284	KP761110
*Rhodotorula mucilaginosa*	5	LSU	EXF-9363	KP761111
*Saprochaete/Magnusiomyces* clade	3	LSU	EXF-8851	KP761118
		ITS	EXF-8851	KP761158
**Wall**				
*Candida parapsilosis*	3	LSU	EXF-9106	KP761092
*Cryptococcus albidus*	1	LSU	EXF-9228	KP761099
*Cryptococcus liquefaciens*	1	LSU	EXF-9230	KP761101
*Exophiala dermatitidis* genotype A	2	ITS	EXF-9138	KP761129
*Exophiala dermatitidis* genotype B	1	ITS	EXF-9367	KP761137
*Exophiala phaeomuriformis* genotype 1	2	ITS	EXF-8888	KP761146
*Saprochaete/Magnusiomyces* clade	1	LSU	EXF-8857	KP761119
		ITS	EXF-8857	KP761159
**Side nozzle**				
*Aspergillus* section *Fumigati*	1	*benA*	EXF-9170	KP761173
*Candida parapsilosis*	1	LSU	EXF-9206	KP761093
*Clavispora lusitaniae*	1	LSU	EXF-9678	KP761098
*Exophiala dermatitidis* genotype A	7	ITS	EXF-9393	KP761130
*Exophiala dermatitidis* genotype B	1	ITS	EXF-9365	KP761138
*Exophiala dermatitidis* genotype C	1	ITS	EXF-8954	KP761142
*Exophiala phaeomuriformis* genotype 1	5	ITS	EXF-9028	KP761147
FDSC	3	ITS	EXF-9176	KP761154
*Meyerozyma guilliermondii*	1	LSU	EXF-9204	KP761105
*Saprochaete/Magnusiomyces* clade	5	LSU	EXF-8856	KP761120
		ITS	EXF-8856	KP761160
		LSU	EXF-9492	KP761115
		ITS	EXF-9492	KP761161
		LSU	EXF-9659	KP761116
		ITS	EXF-9659	KP761162
**Door**				
*Aureobasidium melanogenum*	2	ITS	EXF-9540	KP761125
*Candida parapsilosis*	9	LSU	EXF-9370	KP761094
*Candida zeylanoides*	1	LSU	EXF-9682	KP761097
*Cryptococcus diffluens*	2	LSU	EXF-9068	KP761100
*Cystobasidium slooffiae*	1	LSU	EXF-9107	KP761102
*Debaryomyces hansenii*	1	LSU	EXF-9069	KP761103
*Exophiala dermatitidis* genotype A	4	ITS	EXF-9256	KP761131
*Exophiala dermatitidis* genotype B	1	ITS	EXF-9309	KP761139
*Meyerozyma guilliermondii*	5	LSU	EXF-9279	KP761106
*Penicillium crustosum*	1	*benA*	EXF-9444	KP761174
*Rhodotorula mucilaginosa*	2	LSU	EXF-9310	KP761112
**Rinse-aid dispenser**				
*Candida parapsilosis*	1	ITS	EXF-9275	KP761127
*Exophiala dermatitidis* genotype B	1	ITS	EXF-9290	KP761140
*Exophiala phaeomuriformis* genotype 1	1	ITS	EXF-9125	KP761148
FDSC	2	ITS	EXF-9174	KP761155
*Saprochaete/Magnusiomyces* clade	1	LSU	EXF-9056	KP761117
		ITS	EXF-9056	KP761163
**Sprinkler**				
*Exophiala dermatitidis* genotype A	1	ITS	EXF-9345	KP761132
*Exophiala phaeomuriformis* genotype 1	1	ITS	EXF-9134	KP761149
FDSC	1	ITS	EXF-9171	KP761156
*Pichia kudriavzevii*	1	LSU	EXF-9343	KP761108
**Detergent dispenser**				
*Aureobasidium melanogenum*	1	ITS	EXF-9516	KP761126
*Exophiala dermatitidis* genotype A	1	ITS	EXF-9522	KP761133
*Exophiala phaeomuriformis* genotype 1	1	ITS	EXF-9127	KP761150
*Saccharomyces cerevisiae*	1	LSU	EXF-9104	KP761113
*Trametes versicolor*	1	ITS	EXF-9175	KP761168
**Cutlery rack**				
*Candida parapsilosis*	1	LSU	EXF-9096	KP761095
*Exophiala phaeomuriformis* genotype 1	1	ITS	EXF-9130	KP761151
FOSC	1	*tef*	EXF-9335	KP761170
FSSC	1	*tef*	EXF-9327	KP761171
*Saprochaete/Magnusiomyces* clade	2	ITS	EXF-8933	KP761164
*Pichia kudriavzevii*	1	LSU	EXF-8860	KP761109
**Drain**				
*Candida parapsilosis*	1	LSU	EXF-8894	KP761096
*Candida intermedia*	1	LSU	EXF-9685	KP761090
*Exophiala dermatitidis* genotype A	8	ITS	EXF-9455	KP761134
*Exophiala dermatitidis* genotype C	1	ITS	EXF-9035	KP761143
*Exophiala phaeomuriformis* genotype 1	1	ITS	EXF-9124	KP761152
FDSC	3	ITS	EXF-9154	KP761157
*Meyerozyma guilliermondii*	1	LSU	EXF-9047	KP761107
*Saccharomyces cerevisiae*	1	LSU	EXF-9658	KP761114
*Saprochaete/Magnusiomyces* clade	5	LSU	EXF-8852	KP761121
		ITS	EXF-8852	KP761165
		LSU	EXF-9055	KP761122
		ITS	EXF-9055	KP761166
		LSU	EXF-9456	KP761123
		ITS	EXF-9456	KP761167
*Trichosporon coremiiforme*	1	LSU	EXF-8893	KP761124

EXF, Ex Culture Collection of Extremophilic Fungi; LSU, 26S ribosomal RNA gene; ITS, internal transcribed spacer 1, 5.8S ribosomal RNA gene, and internal transcribed spacer 2; *tef*, translation elongation factor 1-alpha (EF1a) gene; *benA*, beta-tubulin gene; FOSC, *Fusarium oxysporum* species complex; FDSC, *Fusarium dimerum* species complex; FSSC, *Fusarium solani* species complex

**Table 3:** Comparison of occurrence (%) of selected opportunistic pathogenic fungi in 9 locations of 30 residential dishwashers, sampled in the present study.

Location in dishwasher	Contamination with fungi	*E*. *dermatitidis* genotype A	*E*. *phaeomuriformis* genotype 1	*C*. *parapsilosis sensu stricto*	*Saprochaete/ Magnusiomyces* clade	*R*. *mucilaginosa*	FOSC	FDSC
Rubber seal	56.7	26.7	13.3	20.0	10.0	16.7	13.3	3.3
Side nozzle	50.0	23.3	16.7	3.3	16.7	0.0	0.0	10.0
Door	46.7	13.3	0.0	30.0	0.0	6.7	0.0	0.0
Drain	43.3	26.7	3.3	3.3	16.7	0.0	0.0	10.0
Wall	23.3	6.7	6.7	10.0	3.3	0.0	0.0	0.0
Cutlery rack	13.3	0.0	3.3	3.3	6.7	0.0	3.3	0.0
Detergent dispenser	13.3	3.3	3.3	0.0	0.0	0.0	0.0	0.0
Rinse-aid dispenser	10.0	0.0	3.3	3.3	3.3	0.0	0.0	6.7
Sprinkler	6.7	3.3	3.3	0.0	0.0	0.0	0.0	3.3

FOSC, *Fusarium oxysporum* species complex; FDSC, *Fusarium dimerum* species complex.

Rubber seals of the dishwashers were the most frequently contaminated with fungi (57%; 17/30 dishwashers), followed by side nozzles (50%; 15/30), door (47%; 14/30) and drain (43%; 13/30). Although all of the sampled sites in the interior of the dishwashers were contaminated with fungi, the wall (23%; 7/30), sprinkler (7%; 2/30), cutlery rack (13%; 4/30), detergent dispenser (13%; 4/30), and rinse-aid dispenser (10%; 3 /30) were less affected (Table 4).

**Table 4:** Occurrence (%) of selected opportunistic pathogenic fungi in kitchens without and with dishwashers.

Location in kitchen	Kitchen with dishwasher	Presence of fungi (%)	*E*. *dermatitidis* A, B, C (%)	*E*. *phaeomuriformis* genotype 1 (%)	*C*. *parapsilosis* (%)	*A*. *melanogenum* (%)	*R*. *mucilaginosa* (%)	FOSC (%)	FDSC (%)
Sink	No	78.6	0.0	14.3	35.7	0.0	21.4	7.1	0.0
	Yes	46.7	13.3	3.3	26.6	6.6	13.3	0.0	0.0
Drain	No	85.7	0.0	28.6	42.8	7.1	35.7	7.1	0.0
	Yes	76.7	6.6	3.3	50.0	6.6	30.0	6.6	6.6
Rubber drain	No	50.0	0.0	14.2	21.4	7.1	21.4	7.1	0.0
	Yes	53.3	6.6	13.3	20.0	3.3	40.0	3.3	3.3
Dish rack	No	85.7	7.1	14.2	35.7	0.0	50.0	7.1	0.0
	Yes	60.0	6.6	20.0	23.3	10.0	23.3	23.3	6.6
Counter	No	50.0	0.0	0.0	21.4	0.0	7.1	7.1	0.0
	Yes	56.7	6.6	3.3	30.0	6.6	23.3	3.3	0.0
Grid on taps	No	14.3	0.0	7.1	0.0	0.0	0.0	0.0	0.0
	Yes	43.3	6.6	10.0	16.6	3.3	16.6	0.0	6.6

FOSC, *Fusarium oxysporum* species complex; FDSC, *Fusarium dimerum* species complex.

Black yeast-like fungi of the genera *Exophiala* (primarily *E*. *dermatitidis*) were the predominant contamination on rubber seals (40%; 12/30 dishwashers) but were less frequently found in/on drain (30%; 9/30), side nozzle (27%; 8/30), door (17%; 5/30), and wall (10%; 3/30). The occurrence of black yeast-like fungi on other sampled sites inside dishwashers, such as sprinkler, cutlery rack and dispensers, did not exceed 6% (Table 3).

Regardless of sampling site, 47% (14/30) of the tested dishwashers were positive for *E*. *dermatitidis* genotype A, 7% (2/30) for *E*. *dermatitidis* genotype B, and 3% (1/30) for *E*. *dermatitidis* genotype C. Although *E*. *dermatitidis* genotype A prevailed on both rubber seals and drains of the same dishwashers (27%; 8/30), it was also found for side nozzle, door, wall, sprinkler, and detergent dispenser of these dishwashers in lower frequencies (Table 3). One strain isolated from a rubber seal belonged to *E*. *dermatitidis* genotype A2. *E*. *dermatitidis* genotype B was isolated from several sites inside two dishwashers (i.e., rubber seal, door, wall, side nozzle, rubber seal on rinse-aid dispenser), while *E*. *dermatitidis* genotype C was detected for only one dishwasher, although in three different sites (i.e., rubber seal, side nozzle, dishwasher drain). This same dishwasher also contained *E*. *dermatitidis* genotype A and C (side nozzles). *E*. *phaeomuriformis* genotype 1 prevailed on side nozzles (17%; 5/30 dishwashers), followed by rubber seal (13%; 4/30), and wall (7%; 2/30). The black yeast *A*. *melanogenum* was isolated only from door (7%; 2/30) and detergent dispenser (3%; 1/30) (Table 4).

*C*. *parapsilosis sensu stricto* occurred most frequently on dishwasher doors (30%; 9/30), rubber seals (20%; 6/30) and walls (10%; 3/30), contamination of all these critical points with *C*. *parapsilosis* was detected in 3 dishwashers. *R*. *mucilaginosa* was isolated only from rubber seals (17%; 5/30) and doors (7%; 2/30) but not in the same dishwashers. Amongst the important human pathogenic fungi, there were also isolates of the *Saprochaete/Magnusiomyces* clade that were isolated from 7 of the dishwashers, which were recovered from side nozzles (17%; 5/30), drain (17%; 5/30), rubber seal (10%; 3/30), cutlery rack (7%; 2/30), wall (3%; 1/30) and rinse-aid dispenser (3%; 1/30). The FDSC was isolated from side nozzles (10%; 3/30), drain (10%; 3/30) and rinse-aid dispenser (7%; 2/30) of the 5 dishwashers. The FOSC prevailed on rubber seals (13%; 4/30) and cutlery rack (3%; 1/30) of the same dishwashers, while the FSSC was isolated from two dishwashers only, from rubber seal (3%) and cutlery rack (3%) (Table 3).

The tube for the water inlet into the dishwasher was colonised with the FDSC, while the drain hose for the disposal of dishwasher waste water was contaminated with *E*. *dermatitidis* genotype A, *C*. *parapsilosis* and the FOSC.

### *Exophiala dermatitidis* does not contaminate the washed dishes

As the washed dishes represent the main contact point between the users of dishwashers and opportunistic pathogenic fungi, 44 washed items were investigated for the presence of fungi immediately after the end of the washing cycle (30 samples) and also after the washing of similar items by hand (14 samples) (Table 1). Eighteen percent of all of the washed items were contaminated with fungi, irrespective of the type of material they were made from. In total, 11 fungal species were isolated, belonging to filamentous genera *Aspergillus*, *Cladosporium*, *Fusarium*, *Phoma* and *Trichoderma*, and two yeast species from the genera *Candida* and *Rhodotorula*. Filamentous fungi prevailed on dishwasher washed items, whereas yeast and filamentous fungi were present on hand-washed items. For instance, the handle of a frying pan that was washed in a dishwasher was contaminated with the FDSC, while a plastic container for food storage that was washed by hand was contaminated with *C*. *parapsilosis*. *E*. *dermatitidis* and other black yeast-like fungi were not detected in any case.

### *Exophiala dermatitidis* prevails in biofilms on dishwasher rubber seals

The analysis of the different sites inside these dishwashers revealed the highest incidence of fungi on rubber seals. Quantification of the fungal contamination per 1 cm^2^ rubber seal was performed for the predominant species of fungi, which were: *E*. *dermatitidis*, *E*. *phaeomuriformis*, *R*. *mucilaginosa* and *C*. *parapsilosis sensu stricto*. *E*. *dermatitidis* strongly prevailed over the other fungi, with levels from 10^2^ CFU per cm^2^ to a maximum of 10^6^ CFU per cm^2^. The levels of *E*. *phaeomuriformis* and *R*. *mucilaginosa* were similar and also high, in the range from 10^3^ CFU per cm^2^ to a maximum of 10^5^ CFU per cm^2^, while *C*. *parapsilosis sensu stricto* did not exceed 10^2^ CFU per cm^2^.

### Variation of composition of the fungal biofilm on rubber seals with water availability

**Fig 3:**
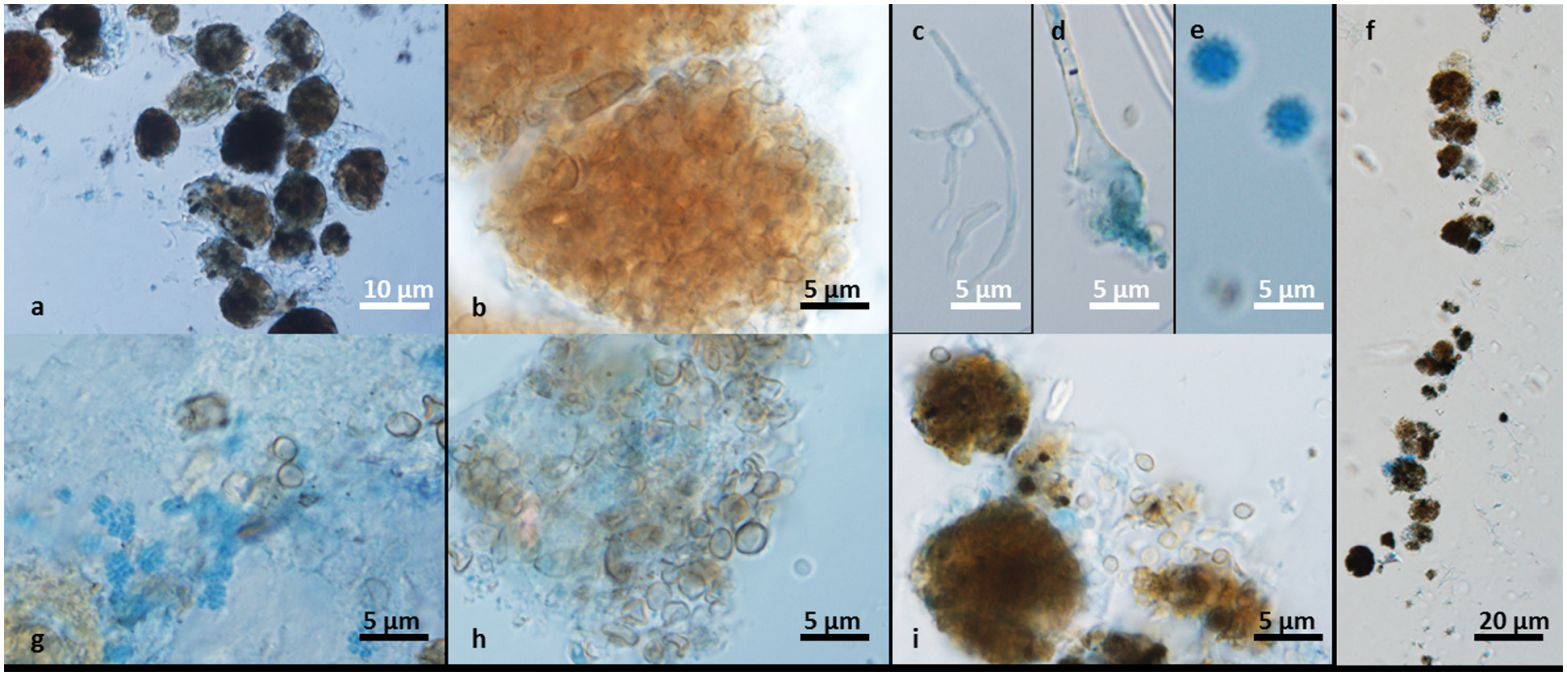
Representative samples from biofilm formed on dishwasher rubber seal. **(a-f)** Dehydrated biofilm. **(a, b, f)** *In-vivo* morphology (muriform cells embedded in extracellular polymeric substance (EPS)) from a biofilm on a dried rubber dishwasher seal from which *E*. *dermatitidis* genotypes A and C were isolated. **(c-e)** Structures of filamentous fungi after desiccation show *Fusarium* spp. **(c)**, *Aspergillus* spp. **(d)** and spores **(e)**. **(g-i)** Hydrated biofilm. Co-culture of bacteria and fungi **(g)**, clusters of yeast cells covered with EPS, with additional individual cells that spread during the washing cycle **(h)**, and muriform cells of *E*. *dermatitidis* genotype A with fungal hyphae **(i)**.

**Fig 4:**
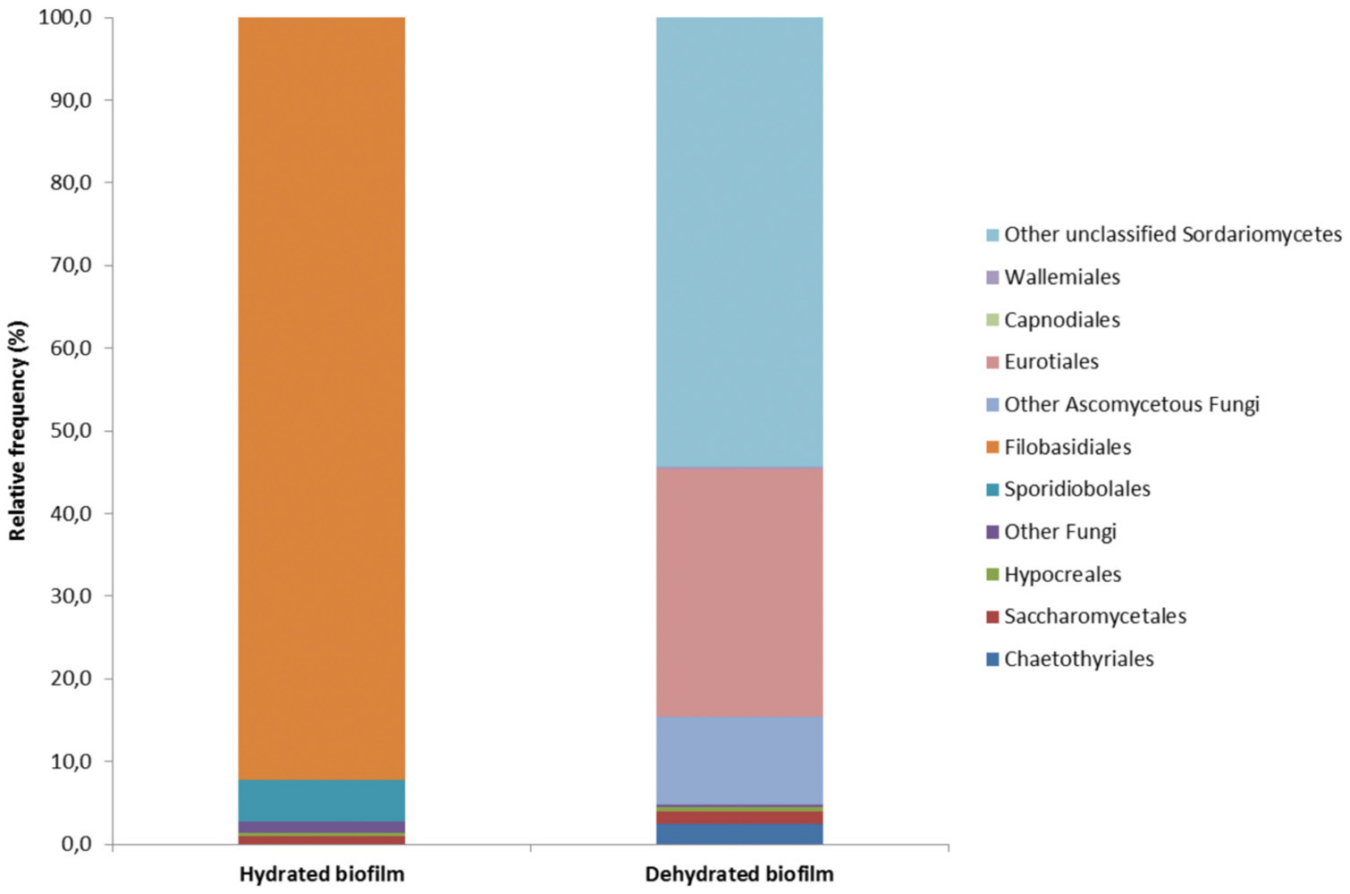
Orders of fungi identified from the hydrated and dehydrated biofilms. **Left**: The hydrated biofilm samples contained six different orders of fungi, as mainly *Filobasidiales* (especially genus *Cryptococcus*; *Basidiomycota*), *Sporidiobolales* (genus *Rhodotorula*; *Basidiomycota*) and *Saccharomycetales* (genus’s *Saccharomyces*, *Hanseniaspora*, *Debaryomyces*; all belong to the *Ascomycota*). **Right**: The dehydrated biofilms contained nine different orders of fungi, as mainly *Hypocreales* (including different moulds and genus *Fusarium*; *Ascomycota*), *Eurotiales* (genus’s *Aspergillus*, *Penicillium*, *Talaromyces*; all belong to the *Ascomycota*) and *Xylariales* (genus *Hansfordia*; *Ascomycota*). Both hydrated and dehydrated biofilms contained *Chaerothyriales* (*E*. *dermatitidis*) (0.07%, 1.77%, respectively) and *Sordariomycetes* (*F*. *dimerum*) (0.3%, 0.35%, respectively).

The fresh, hydrated biofilm samples were seen to be more diverse than the dehydrated, old biofilm samples (Figs 3 and 4). The hydrated biofilm included 16,670 reads assigned to OTUs, with equal levels of *Ascomycota* and *Basidiomycota*, while the dehydrated biofilm included 4,846 OTUs, dominated by *Ascomycota*. The hydrated biofilm contained markedly more yeast, such as *Filobasidiales* (*Cryptococcus*), *Sporidiobolales* (*Rhodotorula*) and *Saccharomycetales* (*Debaryomyces*, *Hanseniaspora*, *Saccharomyces*), while the dried biofilm contained mainly filamentous *Hypocreales* (*Fusarium*), *Eurotiales* (*Aspergillus*, *Penicillium*), and black yeast-like *Chaetothyriales* (*Exophiala*, *Phialophora*).

At the species level, *E*. *dermatitidis* and *F*. *dimerum* were present in the hydrated and dehydrated biofilms, as 12 OTUs (0.07%) and 86 OTUs (1.77%) for *E*. *dermatitidis*, and 50 OTUs (0.30%) and 17 OTUs (0.35%) for *F*. *dimerum*. The raw sequence data have been submitted to the NCBI SRA database under accession No. SRP059618 (NCBI BioProject PRJNA287303).

### *Exophiala dermatitidis* and other fungi are released from biofilms into washing water and waste water

A selected dishwasher with a known contamination of *E*. *dermatitidis* genotype A on rubber seal (10^3^ CFU/cm^2^) and also with *E*. *dermatitidis* on door and dishwasher drain was monitored for the release of *E*. *dermatitidis* into the water during the washing cycle. After 30 min of the washing cycle, the total number of all detected fungi did not exceed 50 CFU/mL in the water. After 1 h, the fungi in the water peaked at 300 CFU/mL, and then dropped in the water of the final wash, to <10 CFU/mL fungi. A similar pattern was observed for the total number of *E*. *dermatitidis*: after 30 min of the washing cycle, there was <5 CFU/mL *E*. *dermatitidis* in the water, after 1 h these levels increased to 50 CFU/mL, and before the end of the washing cycle this had dropped to 2 CFU/mL *E*. *dermatitidis*. No differences in the fungal levels were seen between the water flowing over the dishes and the water collected at the bottom of the dishwasher.

Examination of the waste water from seven dishwashers randomly selected from the 30 included revealed that five of these dishwashers (71%; 5/7) were positive for fungi. These fungi in the waste waters contained exclusively different species of *Exophiala* and *Sarocladium killiense*: *E*. *dermatitidis* was identified for 60% of the isolates and *E*. *oligosperma* for 20%, while the remaining 20% were co-cultures of *E*. *oligosperma* and *S*. *killiense*.

### Hot aerosols released from dishwashers at the end of the washing cycle contribute to dispersal of fungi on kitchen surfaces

Dishwashers are often opened before the end of the cooling process, which can result in the release of hot aerosols. As these might serve as fungal vectors for transmission into the kitchens, we sampled these hot aerosols from 29 of the dishwashers for the presence of fungi (Table 1). Although the analysis revealed the prevalence of ubiquitous air-borne filamentous *Aspergillus* spp. (83%; 24/29), *Penicillium* spp. (55%; 16/29), *Trichoderma* sp. (21%; 6/29) and *Cladosporium* sp. (21%; 6/29), yeast were also present. They were represented by *R*. *mucilaginosa* (7%; 2/29) and *C*. *parapsilosis sensu stricto* (14%; 4/29).

*E*. *dermatitidis* was not detected by cultivation in 100 L of aerosol; however, when a Coriolis cyclone collector was used to sample 1,000 L of aerosol and a specific primer set for the detection of *E*. *dermatitidis* was used, its presence in the aerosol was confirmed.

### Kitchens with a dishwasher have higher incidence of human opportunistic black yeast than kitchens without a dishwasher

Dishwashers provide an extreme environment that can favour the selection and enrichment of certain opportunistic fungi, and particularly thermophilic black yeast. Thus, we assumed that the fungal burden in the 30 kitchens with dishwashers that were sampled would differ from 14 kitchens without dishwashers that were sampled (Fig 2). The data here show that irrespective of the sampling site, all of the kitchens without dishwashers that were sampled were contaminated with fungi, as well as 96% of the kitchens with dishwashers that were sampled. In the kitchens with dishwashers, the most contaminated sites were the kitchen drain (78%; 23/30) and the dish drying rack (60%; 18/30), followed by the kitchen counter (57%; 17/30) and the rubber seal on the kitchen drain (53%; 16/30). Other critical sites for fungal colonisation in these kitchens with dishwashers were the kitchen sinks (47%; 14/30) and the grids on the taps (43%; 13/30). In the 14 kitchens without dishwashers, the most contaminated sites were again the kitchen drain and dish drying rack (both 86%; 12/14), followed by the kitchen sink (78%; 11/14), the kitchen counter and the rubber seal on the kitchen drain (both 50%; 7/14), and the rubber seals on the taps (14%; 2/14). The diversity of the fungal species detected was greater in the kitchens with dishwashers than for those without (53 *versus* 27 fungal species, respectively) (Table 1). Also, in the kitchens with dishwashers, black yeast-like fungi of the genera *Exophiala*, *Aureobasidium*, *Cladophialophora*, *Fonsecaea* and *Ochroconis* were detected. These were isolated from the dish drying racks (37%; 11/30), the rubber seals on the kitchen drains (27%; 8/30), the kitchen sinks and the grids on the kitchen taps (both 23%; 7/30), followed by the kitchen drain (20%; 6/30) and the kitchen counter above the dishwasher (13%; 4/30). Although the proportion of the different detected species varied between the sampling sites, *E*. *dermatitidis* predominated. In the kitchens without dishwashers, these previously listed genera of black yeast were isolated as well, in particular from kitchen drains (43%; 6/14), dish drying racks (36%; 5/14) and kitchen sinks (21%; 3/14), with the exception of *E*. *phaeomuriformis*, which showed much lower quantities (Table 4). Thus, the most important difference between the kitchens without and with dishwashers was significantly lower contamination of kitchens without dishwashers with black yeast, except for *E*. *phaeomuriformis*, and a strong prevalence of white yeast, in particular *C*. *parapsilosis* (Table 4). For the kitchens without dishwashers, the sites that were most heavily colonised with *C*. *parapsilosis* were the kitchen drain (43%; 6/14), the kitchen sink and the dish drying rack (both 36%; 5/14), the rubber seal on the kitchen drain, and the kitchen counter near the sink (both 21%; 6/14). For the kitchens with dishwashers, the sites that were most contaminated with *C*. *parapsilosis* were the kitchen drain (50%; 15/30), the kitchen counter above the dishwasher (30%; 9/30) and the kitchen sink (27%; 8/30).

**Fig 5:**
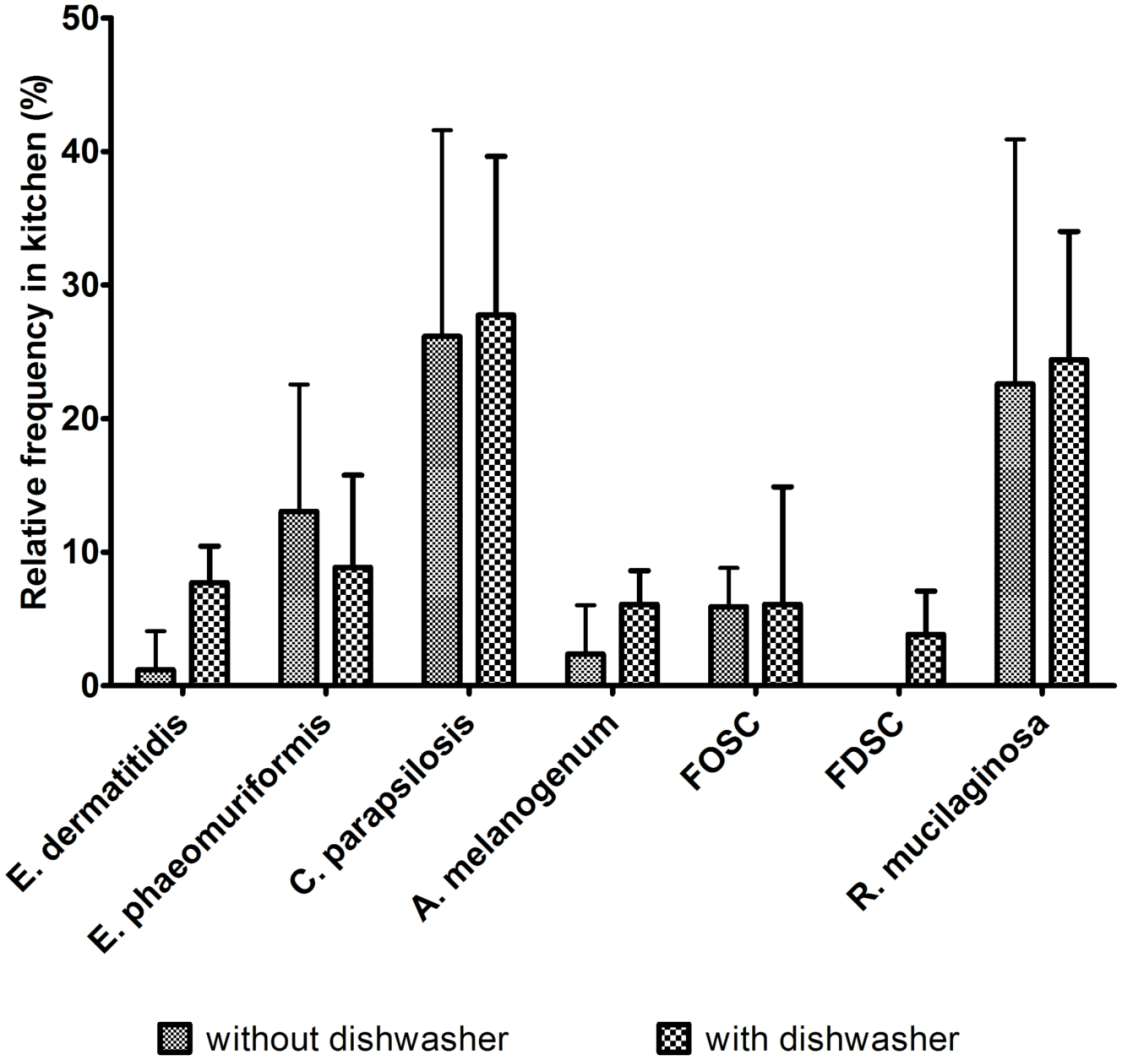
Relative mean percentages (±standard deviation) of the seven opportunistic fungal pathogens most commonly isolated from the kitchen surfaces.

The follow-up of the dishwasher mycobiota in the kitchens (Fig 5) revealed that for those kitchens with dishwashers, the highest incidence of *E*. *dermatitidis* was inside the dishwashers (47%; 14/30), in particular on rubber seals, followed by the different surfaces (23%; 7/30). The occurrence of *E*. *dermatitidis* in the kitchens without dishwashers was almost negligible (7%; 1/14). *E*. *phaeomuriformis* was present in 30% of tested dishwashers (9/30), mainly on side nozzles (17%; 5/30) and in both types of kitchen, however in 4% (3/14) higher incidence in kitchens without dishwashers. *A*. *melanogenum* was present in the tap water in 16% (7/44) of the samples, which was reduced in the dishwashers to 7% (2/30), but increased in frequency to 23% (7/30) in the kitchens with dishwashers, and to 14% (2/14) in kitchens without dishwashers. *C*. *parapsilosis* was detected in 11% (5/44) of the tap water samples, which increased to 30% (9/30) in the dishwashers, and it was also found in the contaminated kitchens with a dishwasher at a higher percentage (77%; 23/30) than in kitchens without a dishwasher (64%; 9/14). *R*. *mucilaginosa* colonised 23% of the dishwashers (7/30), which increased to 70% in kitchens with a dishwasher (21/30) and to 79% in kitchens without a dishwasher (11/14). The FOSC was observed inside dishwashers (23%; 7/30), in kitchens with a dishwasher at 17% (5/30), whereas there were no FOSC isolates obtained from kitchens without a dishwasher (Fig 5). Species complex *Saprochaete/Magnusiomyces* was detected only inside dishwashers (27%; 8/30), and none of these isolates were obtained from kitchens either without or with dishwashers.

## Discussion

Ever since the first human dwellings emerged approximately 20,000 years ago [38], the indoor biome has been evolving [39]. Fast economic growth and increased housing standards have changed human houses most dramatically during the last few decades. Modern indoor biomes are much less influenced by outdoor changes, have easy access to water, and contain an abundance of synthetic materials and household appliances that facilitate our daily routines. Although humans and microbes have always co-existed in indoor biomes, modern developments have brought many changes to this coexistence. The most successful of the indoor microbes have adapted to fluctuating stresses, and can degrade different man-made materials, grow as biofilms on different inanimate surfaces [40–42], and resist increased sanitation and hygienic measures and abundant amounts of antimicrobials [43]. These conditions have enriched for metabolically versatile, polyextremotolerant microbial species, with increased pathogenic potential [42,44,45]. This provides a worrisome perspective for human health in the light of numerous opportunities for selected/ evolved pathogenic species to come into contact with humans who are now generally more susceptible to infections in comparison to the past [2].

Studies on indoor pathogenic microbes have so far been mostly limited to bacteria, and have shown that kitchens and bathrooms are the most contaminated parts of the house [41,46]. Thus, these rooms can become reservoirs of *Mycobacteria* [47,48], *Legionella* spp., and *Pseudomonas aeruginosa* [49]. With respect to fungi, research has mainly focused on their presence in indoor air and on contaminated food [50,51]. Only recently, it became clear that opportunistic pathogenic extremophilic black fungi invade domestic bathrooms [10,52], drainpipes that are warmed daily to over 42°C [53], steam baths [54], sink drains [11], drinking water [55], and even washing machines [56] and dishwashers [20,21]. So far fungal contamination of dishwashers was reported only on rubber seals [20, 21], without further investigation into the fungal colonisation of the dishwasher interior.

Two independent studies [20,21] reported that geographically distant dishwasher rubber seals were contaminated to a large extent by an overlapping species diversity, which represented seven species in particular: the black yeast *E*. *dermatitidis* and *E*. *phaeomuriformis* as the main colonisers, followed by *C*. *parapsilosis*, *M*. *capitatus*, *R*. *mucilaginosa*, *Pichia* (now *Meyerozyma) guilliermondii* and *F*. *dimerum*, in this order. These consistently present “dishwasher mycobiota” represent the “dishwasher microbiome” that reflects selection processes that have occurred in dishwashers.

In the present study, we selected 30 dishwashers that differed in age, frequency of use, location and connection to water supply system. We extended the sampling to include eight additional sites inside the dishwashers that differ in terms of the material (e.g., plastic, steel) and the position with regard to the ejection of water (e.g., side wall, bottom). We also sampled the washed items of different materials (e.g., glass, plastic, porcelain, ceramic, metal). Irrespective of the sampled sites, 83% of the sampled dishwashers were positive for fungi. Although rubber seals again were the most heavily contaminated sites of dishwashers (57%), thigh contamination was detected also on side nozzles, doors and drains (43%-50%). The lowest fungal contamination was observed on the sprinklers and the washed items (both at 7%). The black yeasts were represented mainly by *E*. *dermatitidis*, prevailing at almost all sampled sites. Although *E*. *dermatitidis* was detected most abundantly on rubber seals with cell densities up to 10^6^ CFU/cm^2^, it was also present on all of the other sampled parts (6%-30%), with the exception of the washed and dried items.

Several species of black yeast, including *E*. *dermatitidis*, are known for their metabolism of aromatic pollutants and their formation of biofilms on contaminated materials [40]. Investigation of dishwasher biofilms has revealed colonization by different fungi. The most obvious structures within the biofilm were muriform clusters of viable *E*. *dermatitidis* cells, in hydrated, as well as in completely dehydrated state. The results of the metagenomic analysis of fungal community in the dry biofilm of a 7 years old, but 6 months unused dishwasher, disconnected from water source, resulted in a marked reduction of yeast species. Yeasts are much more sensitive to desiccation than sporulating fungi; spores may remain viable also in very dry environments and resume growth as mycelium when conditions improve.

A distinctive array of phenolic and aliphatic compounds that are products of the degradation of aromatic hydrocarbons by black yeast like fungi, especially *E*. *dermatitidis*, have roles in neurophysiology, since a connection between aromatic hydrocarbon assimilation and certain mammalian neurological infections has been suggested [57,58]. *E*. *dermatitidis* can cause systemic, disseminated neurotropic and brain infections (occurring particulary in East Asia) even in healthy, immunocompetent individuals [59,60]. *E*. *dermatitidis* can also cause cutaneous and subcutaneous infections [61,62], and is the third most frequent respiratory fungal pathogen that infects patients with cystic fibrosis [25]. While *E*. *dermatitidis* has rarely been solated in nature [60,63,64], it dominates dishwashers [20], and is frequently present in bathrooms [11], saunas and steam baths [54]. These ecological preferences might be explained by its ability to degrade aromatic hydrocarbons in combination with its tolerance to high temperatures and rapid changes in pH [20], as well as with its pleomorphic morphology [65]. In the context of its frequent presence in the domestic environment it gives reasons for concern.

The dishwasher mycobiome contains another opportunistic pathogenic *Exophiala* species, namely *E*. *phaeomuriformis*. We found *E*. *phaeomuriformis* to be present on 13% of rubber seals (with settlement index up to 10^5^ CFU/cm^2^), while previous studies have detected it in up to 6% of dishwashers [20,21]. In agreement with its water-borne nature, *E*. *phaeomuriformis* was present at even higher levels on the side nozzles (17%), which eject water side-ways onto the items in the dishwasher. *E*. *phaeomuriformis* forms a stable community on rubber seals together with *E*. *dermatitidis* in 5%-7% of the observed cases. Cocultures of these black yeasts were detected additionally on the side nozzles (10%) and the dishwasher sprinklers (3%). *E*. *phaeomuriformis* has so far been described solely as a coloniser of bathrooms [54], and similar to *E*. *dermatitidis*, it has been isolated from nature extremely rarely. According to the CBS Culture Collection database (http://www.cbs.knaw.nl/Collections; March 2015), to date only one *E*. *phaeomuriformis* strain has been isolated in nature, from a natural hot spring (CBS 109150). *E*. *phaeomuriformis* grows at a lower temperature range than *E*. *dermatitidis* [54] and it is not covered by a capsule [66]; however, it is involved in similar medical conditions as *E*. *dermatitidis*. *E*. *phaeomuriformis* has been reported in patients with cystic fibrosis [67,68], and in a few cases from severe brain and disseminated infections [67].

One of the newly detected opportunistic pathogenic species of black yeast from dishwashers in the present study was *A*. *melanogenum* [69], previously known as *A*. *pullulans* var. *melanogenum* [70]. *A*. *melanogenum* is an opportunistic pathogenic black yeast that can grow at 37°C, and it is ubiquitous in oligotrophic aqueous environments from the Arctic to the tropics [70]. The water-borne nature of *A*. *melanogenum* is reflected in its high presence in the tap water systems connected to the dishwashers here (16%), however, the conditions inside dishwashers were mainly prohibitive for its spread since it was identified only on doors (7%) and in detergent dispensers (3%).

*Saprochaete clavata* is phylogenetically and phenotypically closely related to *M*. *capitatus*, and it has also been reported as a consistent representative of the dishwasher mycobiota [20,21]. Most *M*. *capitatus* dishwasher isolates reported by Zalar et al. (2011) [20] were reidentified by Vaux et al. (2014) [71] as *S*. *clavata*, and one as *M*. *capitatus*. Due to this common misidentification of these two species based on ITS sequences, isolates obtained in the present study were defined as the *Saprochaete/Magnusiomyces* clade [72,73]. The present study not only confirmed the presence of the *Saprochaete/Magnusiomyces* clade on rubber seals (10%), but also detected it on cutlery rack, rinse-aid dispenser, wall, side nozzle and drain (3%-17%). In one of the dishwashers, there was massive dissemination of the *Saprochaete/Magmusimyces* clade on all of the sampled sites of the dishwasher. Both *M*. *capitatus* and *S*. *clavata* are not commonly encountered in the environment. *M*. *capitatus* was isolated once only from heated woodpulp [74], while all of the remaining known strains of *M*. *capitatus* originate from human patients with haematological malignancies [72,75]. The outbreaks of *M*. *capitatus* have been mainly in Europe, primarily related to contaminated dairy products [76–78]. *S*. *clavata* has also been reported as a cause of infection associated with dairy products [77,79,80], up to a recent outbreak in 2012 in France that revealed potential infections via contaminated medical devices [71]. Orally ingested strains can cause diarrhoea associated with fungemia with up to 80% mortality [71]. Its consistent presence in dishwashers is therefore a cause for concern.

The opportunistic white yeast *C*. *parapsilosis* is another consistent member of the dishwasher community on rubber seals [20,21]. However, in this study it was present with a higher frequency on dishwasher doors (30%) than on rubber seals (20%), and recently it has also been reported to be present in washing machines [81]. It has been occasionally isolated from sugary enviroments such as fruit [82,83], and from raw water sources [84]. Otherwise it has been primarily associated with mammals and wild birds, either as an opportunist or as an intestinal coloniser [82,83]. After *C*. *albicans* and *C*. *tropicalis*, *C*. *parapsilosis* is the third most common cause of candidemias in patients [85]. It is involved in 23% of bloodstream infections [86]. *C*. *parapsilosis* infections are mainly associated with contaminated prosthetic devices, indwelling catheters, and the hands of healthcare workers [87,88]. Individuals at highest risk for severe infection include neonates, diabetic and/or elderly people, and patients in intensive care units [86,89].

*Rhodotorula* species have emerged in the last decade as opportunistic pathogens, and they have been reported to cause up to 2.3% of fungemias in the USA [90] and Europe [91]. In most cases, infections were associated with contamination of hands of hospital employees and of patients [92] and with central venous catheters in patients with haematologic malignancies [90,93–95]. *R*. *mucilaginosa* is otherwise a ubiquitous, saprophytic yeast [96] that has been commonly isolated from fruit [97] and meat products [98], and also from aquatic hypersaline and high-temperature environments [99,100]. *R*. *mucilaginosa* was present in diswashers on rubber seals (17%) and on dishwasher doors (7%), where it may come into contact with the user.

The filamentous *F*. *dimerum* is an opportunistic pathogen that was detected on dishwasher rubber seals also in the present study. However, in agreement with its water-born nature [55], here *F*. *dimerum* contaminated primarily sites directly exposed to water, such as drains, sprinklers and side nozzles. *F*. *dimerum* was also the only human opportunistic fungus that was isolated directly from washed dishes. As well as *F*.*dimerum*, this study also found representatives of both the FSSC and the FOSC, which have been reported to cause 80% of all human fungal infections [4]. The FOSC was isolated from rubber seals (13%) and from cutlery racks (3%), while the FSSC was isolated from the same interior sites, although at lower frequencies. The FOSC was previously revealed as the dominant filamentous fungus in 23% of washing machines [56]. Indoor-plumbing-associated biofilms formed by *Fusarium* sp. have been reported as potential sources of infection in hospitals and in the community [13], and might also be the source of contamination of both dishwashers and washing machines as reflected in its presence in the pipes of the inlet plumbing and in the drains.

Few investigations have explored the origins of indoor microbial species and their dispersal in the indoor biome [18,101,102]. It appears that microbes enter houses on humans and their pests via food or with air or water [9,18]. The dependence of many indoor species on passive dispersal results in a stochastic composition of species in any particular indoor biome [101]. In comparison to bacteria, fungi have shown little direct influence of humans, and have shown geographic structures on a global [17] and even local scale [18]. Dishwasher mycobiota can be transmitted into the enclosed extreme habitat of the dishwashers via the water, or through food remains and humans. To segregate water-borne species from those that originate from other sources, we investigated fungal diversity in the tap-water system connected to the sampled dishwashers. With the exception of the *Saprochaete*/*Magnusiomyces* clade, all species that represent the dishwasher mycobiota were detected in the present study in the tap water, and were also previously reported as contaminants of raw water sources and tap water from residential houses [84,103,104]. Although many different fungal species were isolated from incoming water, after enrichment in dishwashers, only *E*. *dermatitidis* (43%), *E*. *oligosperma* (29%) and *S*. *killiense* (14%) were released into dishwasher waste water.

The dishwasher mycobiota can potentially be spread indoors also via aerosols. Although the fungi isolated from aerosols were mainly different species of the common air-borne genera *Aspergillus* (83%), *Penicillium* (55%) and *Cladosporium* (21%) [105–109], these aerosols also contained yeasts. Sowiak at al [107] reported that indoor bioaerosols can contain up to 10% yeast mainly represented by the genus *Candida*, while the aerosols sampled in the present study contained up to 14% *C*. *parapsilosis* and 7% *R*. *mucilaginosa*. Although no black yeast were detected under the conditions tested here, the presence of *E*. *dermatitidis* was confirmed in air sample from 1,000 L of hot steam using *E*. *dermatitidis* specific primers [34]. This is the first report on the occurrence of *E*. *dermatitidis* in air indicating that it can act as a potential vector for the spread of *E*. *dermatitidis* onto kitchen surfaces, and for causing pulmonary infections in humans, which is particularly relevant for patients with cystic fibrosis [25]. Aerosolisation of fungi from water systems can also have other implications for human health [110].

The spread of these opportunistic pathogenic fungi of the dishwasher mycobiota into kitchens where food is processed is a cause for concern. Such fungi can gain access to humans by skin maceration [52], by inhalation, or by ingestion of contaminated food or drink [111]. Although many indoor fungal species show low dispersal [13–15], the colonisation of kitchens might be facilitated by fungal pre-adaptation to local selection pressures, such as within dishwashers. In the present study, we monitored the distribution of fungi in kitchens without and with dishwashers. Virtually all of the sampled kitchens were positive for fungi. Kitchens with dishwashers were populated with a more diverse fungal community that was dominated by black yeasts, which particularly prevailed on man-made materials such as plastic racks for drying dishes, and rubber seals on kitchen drains. The prevailing black yeast that contaminated kitchens with dishwashers was *E*. *dermatitidis*, that also was found in the tap-water system, the dishwasher waste water, and in hot aerosols. The frequency of *E*. *dermatitidis*, was negligible in 14 kitchens without dishwashers.

In contrast, *C*. *parapsilosis* dominated all of the sampled surfaces in kitchens without dishwashers. *C*. *parapsilosis* was isolated not just from kitchen drains, but also from kitchen sinks, neighbouring counters, and dish drying racks. *R*. *mucilaginosa* has so far been reported in bathrooms [108], while this study revealed its frequent presence also on different kitchens surfaces. *Fusarium* spp. have been recovered in many surveys of tap-water systems [112,113], in kitchens [15], and in bathroom drains [13]. The present study detected the FOSC and the FSSC in kitchens both without and with dishwashers; however, these were more frequent in kitchens with dishwashers. They were the only species from the dishwasher mycobiota that remained on the washed dishes. Notably, the FDSC was strikingly found exclusively in kitchens with dishwashers suggesting that the presence of a dishwasher mycobiota is a prerequisit for the occurrence of some fungi in kitchens.

The *Saprochaete/Magnusiomyces* clade represents the only species of the dishwasher mycobiota that were detected only inside the dishwashers and that did not establish themselves in kitchens. They might enter dishwashers via contaminated dairy products on dishes [77,79,80], but they do not spread elsewhere in the indoor biome.

Seventy-three percent of kitchens with dishwashers were contaminated with black yeasts, in comparison with 64% of kitchens without dishwashers. The main difference was observed on the level of black yeast species diversity. Kitchens with dishwashers and dishwashers had a similar composition of black yeasts (Table 1), with the predominance of the genus *Exophiala*, while black yeasts in kitchens without dishwashers were more diverse. We assume that black yeasts, particularly *E*. *dermatitidis*, are being released continuously, at low rate from the dishwashers into the kitchen environment.

Studies of indoor biomes are at the intersection of evolutionary ecology, anthropology, interior design, and human ecology. Our indoor environments are prohibitive for growth of the majority of microbes. However, some species have succeeded in surviving, adapting and spreading within the indoor biome. While for some traits (e.g., antibiotic resistance) this is already widely accepted as a reason for concern, we are still not aware of the other consequences of this selection process that is going on around us, and accentuated by green technologies and decreased energy consumption that result in low temperatures within appliances and using of biodegradable detergents. We might be turning our homes into microcosms for evolution of the most resilient of microbial species, which might in the future find new niches indoors, and in the human body to the detriment of human health.

## Conclusions

Modern indoor biomes provide favourable environments for colonisation and growth of metabolically versatile and resilient microorganisms. Previous studies have focused on pathogenic and antibiotic-resistant bacteria in kitchens and bathrooms. However, with the exception of air-borne fungi causing respiratory diseases and allergies, fungi have been generally neglected. Fungal colonisation of indoor wet areas, such as bathrooms and kitchens, dishwashers and washing machines, has become evident only recently. The fungi that contaminate these environments are mainly selected species of human opportunistic black, white and red yeasts as well as some selected species of filamentous fungi.

We report here that dishwashers are populated primarily by six species: *E*. *dermatitidis*, *C*. *parapsilosis sensu stricto*, *E*. *phaeomuriformis*, *R*. *mucilaginosa*, FDSC and the *Saprochaete/Magnusiomyces* clade. The dominant coloniser is the opportunistic pathogenic black yeast *E*. *dermatitidis* genotype A, rarely isolated from nature, but mainly associated with patients with cystic fibrosis or as a causative agent of neurotropic, cutaneous and subcutaneous infections. *E*. *dermatitidis* contaminates dishwasher interiors in mixed microbial biofilms with up to 10^6^ CFU/cm^2^. *E*. *dermatitidis* probably enters dishwashers via the tap-water system, where we have detected it in low levels. It exits dishwashers via the waste water, hot dishwasher aerosols released at the end of the washing cycle, and contact with contaminated dishwasher surfaces. *E*. *dermatitidis* dominates kitchen surfaces in kitchens with dishwashers, while its presence in kitchens without dishwashers is negligible. A similar pattern was observed for filamentous *F*. *dimerum*, which was the only fungal species isolated from the washed items in the dishwashers. Kitchens without dishwashers were dominated by *C*. *parapsilosis* and *E*. *phaeomuriformis* which were also found in the kitchens with dishwashers, although at lower levels. The *Saprochaete*/*Magnusiomyces* species complex was consistently isolated from dishwashers while it has been extremely rarely isolated from nature and was not found in kitchens either without or with dishwashers. Opportunistic pathogenic black yeasts are present within dishwashers, as well as outside dishwashers on other kitchen surfaces. The occurrence of these species in kitchens without dishwasher is almost negligible.

We conclude that some human opportunistic pathogens, while rare in nature, are enriched and proliferating inside domestic dishwashers. Some of these species additionally spread into kitchens from where they represent and additional threat to human health. To counter this growing danger, it is necessary to study how the establishment of reservoirs of pathogenic fungal strains in dishwashers and other household appliances can be prevented.

## Ethic Statement

In this study, field sampling was performed, and to our knowledge, no endangered or protected species were involved. All of the strains studied here were obtained from the discussed sampling areas, for which permission was obtained from the owners.
